# Decorin inhibits the insulin-like growth factor I signaling in bone marrow mesenchymal stem cells of aged humans

**DOI:** 10.18632/aging.202166

**Published:** 2020-11-26

**Authors:** Tze-Hong Wong, Ting-Yu Chen, Kuo-Yun Tseng, Zih-Ying Chen, Chung-Hsing Chen, Feng-Huei Lin, Hung-Ming Wu, Shankung Lin

**Affiliations:** 1 Department of Orthopedics, National Taiwan University Hospital, Hsin-Chu Branch, Taiwan, Republic of China; 2 Inflammation Research and Drug Development Center, Changhua Christian Hospital, Taiwan, Republic of China; 3 Institute of Infectious Diseases and Vaccinology, National Health Research Institutes, Taiwan, Republic of China; 4 Institute of Population Health Sciences, National Health Research Institutes, Taiwan, Republic of China; 5 Taiwan Bioinformatics Institute Core, National Health Research Institutes, Taiwan, Republic of China; 6 Institute of Biomedical Engineering, College of Medicine and College of Engineering, National Taiwan University, Taiwan, Republic of China; 7 Institute of Biomedical Engineering and Nanomedicine, National Health Research Institutes, Taiwan, Republic of China; 8 Department of Neurology, Changhua Christian Hospital, Taiwan, Republic of China; 9 Graduate Institute of Acupuncture Science, China Medical University, Taiwan, Republic of China; 10 Graduate Institute of Biomedical Sciences, China Medical University, Taiwan, Republic of China

**Keywords:** aging, osteoporosis, IGF-I, small leucine-rich proteoglycan, bone marrow mesenchymal stem cell

## Abstract

Aging impairs the IGF-I signaling of bone marrow mesenchymal stem cells (bmMSCs), but the mechanism is unclear. Here, we found that the ability to auto-phosphorylate IGF-I receptor (IGF-IR) in response to IGF-I was decreased in the bmMSCs of aged donors. Conversely, data showed that decorin (DCN) expression was prominently increased in aged bmMSCs, and that under IGF-I treatment, DCN knockdown in serum-starved aged bmMSCs potentiated their mitogenic activity and IGF-IR auto-phosphorylation, whereas DCN overexpression in serum-starved adult bmMSCs decreased both activities. Co-immunoprecipitation assays suggested that IGF-I and DCN bound to IGF-IR in a competitive manner. Online MethPrimer predicted 4 CpG islands (CGIs) in the introns of *DCN* gene. RT-qPCR and bisulfite sequencing showed that dimethyloxalylglycine, an inhibitor of DNA demethylation, increased *DCN* mRNA expression and CGI-I methylation in adult bmMSCs, whereas 5-aza-2’-deoxycytidine, a DNA methylation inhibitor, decreased *DCN* mRNA expression and CGI-I methylation in aged bmMSCs, and ultimately enhanced the proliferation of serum-starved aged bmMSCs under IGF-I stimulation. Thus, IGF-IR could be the prime target of aging in down-regulating the IGF-I signaling of bmMSCs, where DCN could be a critical mediator.

## INTRODUCTION

Compromise in the functions of organs due to the loss of tissue homeostasis is a general feature among aged population [1]. Tissue homeostasis is supported by the replacement of the aged and damaged cells by plenty of healthy and functional cells derived from the stem/progenitor cells, i.e., the maintenance of tissue cellularity. Loss of cellularity is common in the aging-related diseases. In fact, loss of immune cells increases cancer incidence, while loss of muscle cells and bone-forming cells cause sarcopenia and osteoporosis, respectively. This pathological evidence reflects a causative role of stem/progenitor cells in the development of aging phenotype and aging-related diseases.

Bone marrow mesenchymal stem cells (bmMSCs) is a small population of cells capable of proliferating and differentiating into several types of cells including osteoblast [2–4]. Like the other types of cells, the biological properties of bmMSCs are also modulated by aging. Mounting evidence [5–10] has indicated that aging down-regulates bmMSC’s proliferation rate and osteoblastogenic potential, which results in the loss of osteoblasts in the aged bones. Notably, evidence suggests that down-regulation of the insulin-like growth factor I (IGF-I) signaling may play an important role in the aging of bmMSCs. IGF-I is a mitogen and a mediator of skeletal growth [11, 12]. However, it has been shown that the mitogenic activity of bmMSCs in response to IGF-I was impaired by aging [11, 13], whereas IGF-I overexpression stimulated the proliferation and bone-forming capability of bmMSCs of aged human donors [13]. IGF-I triggers anabolic signals by binding to its cognate receptor, IGF-IR [14]. IGF-I also binds to and activates insulin receptor (IR), but with an affinity 100- to 500-fold lower than that of insulin. The binding of IGF-I results in auto-phosphorylation of IGF-IR at the serine1131 (Ser1131), Ser1135, and Ser1136 residues of its β subunit, and subsequently activates the receptor tyrosine kinase [15], and initiates the corresponding signaling cascades [[16] and the references therein]. Tanaka and Liang examined the expression of IGF-IR and the binding of IGF-I to IGF-IR in bmMSCs of adult and aged rats, and found no significant difference in receptor density and ligand-binding activity [11]. Cao et al. also reported that IGF-I binding was normal in mice regardless of aging [17]. However, how aging impairs the IGF-I signaling of bmMSCs remains unclear. Identification of the intrinsic mediators conferring the inhibitory effect of aging on the IGF-I signaling of bmMSCs will be a plausible approach to elucidate the mechanism.

To gain insights into the basic cause of bone defects in aging, we have previously analyzed the gene expression profiles of bmMSCs isolated from human donors of various age, and retrieved a list of genes whose expression was highly associated with age [5]. Interrogating those age-associated genes using the Ingenuity Pathway Analysis (IPA) has suggested a close link between the cell growth and cell cycle progression, glycosylation, and age. Accordingly, we intended to search for the responsible mediators from those age-associated genes that can regulate the IGF-I signaling of bmMSCs. Here, we provide evidence to support the potential role of decorin (DCN), a proteoglycan in the extracellular matrix [18], as an aging-related IGF-IR inhibitor in bmMSCs.

## RESULTS

### Age-related changes in the mitogenic activity of bmMSCs in response to IGF-I

**Figure 1:**
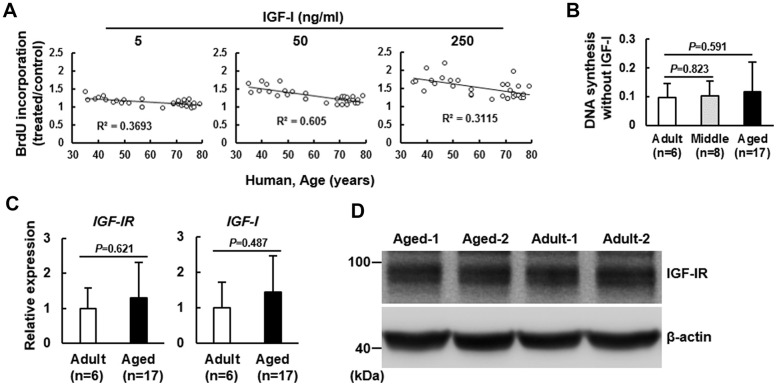
**Effect of age on the mitogenic response of human bmMSCs to IGF-I. BrdU incorporation analyses.** Human bmMSCs were maintained in serum-free media for 24 h, and then subjected to BrdU incorporation analyses with or without concomitant treatment of 5, 50, and 250 ng/ml of IGF-I. (**A**) The correlation between the age and the IGF-I-triggered DNA synthesis was analyzed. (**B**) The average OD_450_ values of bmMSCs of adult (n=6), middle aged (n=8), and aged (n=17) groups cultured without IGF-I treatment are shown. The difference between the groups was analyzed by the Student’s t-test. (**C**) RT-qPCR analyses. The expression of IGF-IR and IGF-I mRNAs of the aged bmMSCs (n=17) were compared to those of the adult bmMSCs (n=6) (to which a value of 1 was assigned). Data represent the mean ± S.D. from three experiments. Student’s t-test was used to analyze the differences between the groups. (**D**) Western blot analyses. The protein levels of IGF-IR in the Aged-1, Aged-2, Adult-1, and Adult-2 cells are shown.

Human bmMSCs were isolated from 6 adult donors (35~43 years), 8 middle age donors (46~57 years), and 17 aged donors (65~79 years). To examine the correlation between age and IGF-I-induced DNA synthesis in bmMSCs, we treated serum-starved bmMSCs with 5, 50, and 250 ng/ml of IGF-I, and performed BrdU incorporation assays. The correlation coefficients between DNA synthesis and age for each dose were -0.6077 (*P*<0.0005), -0.7781 (*P*<10^-6^), and -0.559 (*P*<0.001), respectively, and the IGF-I concentration required for a 50% induction in DNA synthesis was shifted from 50 ng/ml for cells from adult donors to 250 ng/ml for cells from aged donors (Figure 1A). To examine if age decreased BrdU uptake, which might result in underestimate of the DNA synthesis in the aged group, we also examined the BrdU incorporation in these serum-starved bmMSCs without IGF-I treatment, and found that there was no significant difference between adult and middle age groups (*P*=0.823), and between adult and aged groups (*P*=0.591) (Figure 1B). These data suggested that age was unlikely to decrease BrdU uptake by these serum-starved bmMSCs.

To investigate the mechanism underlying the age-related impairment in the IGF-I-induced DNA synthesis, we examined the expression of IGF-I and its receptor (IGF-IR) in the adult and aged bmMSCs. RT-qPCR analyses showed that the expression of *IGF-IR* and *IGF-I* mRNAs in bmMSCs from aged human donors (n=17) was similar to those in bmMSCs from adult donors (n=6) (Figure 1C). Analyses of the IGF-IR protein levels in bmMSCs from randomly selected aged donors (Aged-1 and Aged-2) and adult subjects (Adult-1 and Adult-2) also suggested that IGF-IR expression might not decrease with aging (Figure 1D). Similar results were seen with the use of bmMSCs isolated from Fisher 344 rats with age ranging from 3 to 21 months old (Supplementary Figure 1). Together, these results indicated an age-related impairment in the IGF-I-induced mitogenic activity of bmMSCs. In addition, IGF-IR expression was not down-regulated by aging, and higher doses of IGF-I could substantially increase the DNA synthesis in bmMSCs from aged donors.

### IGF-IR auto-phosphorylation in the age-related impairment of IGF-I signaling

**Figure 2:**
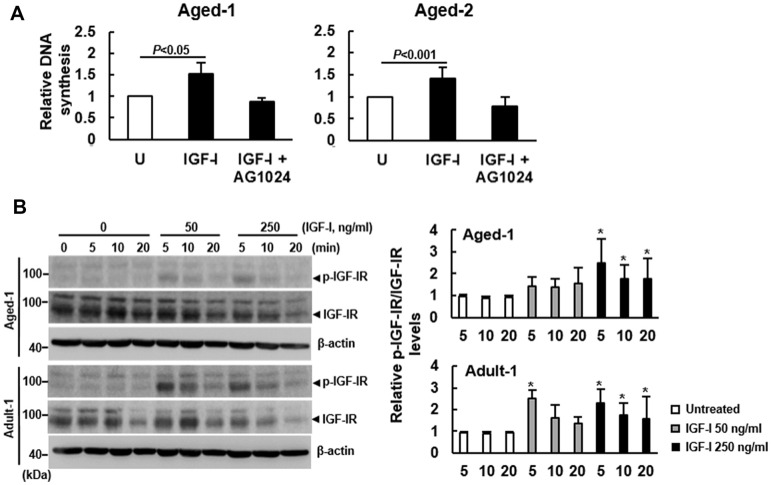
**Effect of aging on the auto-phosphorylation of IGF-IR of bmMSCs.** (**A**) BrdU incorporation analyses. Serum-starved Aged-1 and Aged-2 bmMSCs were examined for the IGF-I-induced DNA synthesis with or without concomitant treatment of AG1024 (1 μM). Relative DNA synthesis was calculated by compared the OD_450_ readings of the treated cells to that of the untreated (U) cells. (to which a value of 1 was assigned). Data represent the mean ± S.D. from three experiments. Student’s t-test was used to analyze the differences between the groups. (**B**) Western blot analyses. Serum-starved Aged-1 and Adult-1 cells were treated with 0, 50, and 250 ng/ml of IGF-I for 0, 5, 10, and 20 min. The auto-phosphorylation of IGF-IR was examined. Representative blots are shown. All the signals were compared to that of the untreated cells at time 0 (to which a value of 1 was assigned). Data represent the mean ± S.D. from three experiments. A one-way ANOVA plus Scheffe’s post hoc tests were used to analyze the differences among the untreated and IGF-I-treated groups. ^*^, *P*<0.05 versus untreated control.

The binding affinity of IGF-I to IGF-IR of murine bmMSCs has been shown not to change with aging [11, 17]. Given our finding that aging did not decrease IGF-IR expression, we set out to examine if aging down-regulated IGF-I-triggered IGF-IR activation. We examined the effect of AG1024, an inhibitor of IGF-IR signaling, on the IGF-I-induced DNA synthesis in Aged-1 and Aged-2 cells. Our data showed that 200 ng/ml of IGF-I caused approximately 52% and 41% increase of DNA synthesis in Aged-1 and Aged-2 bmMSCs, respectively, but the induction was inhibited by the co-treatment of 1 μM of AG1024 (Figure 2A). Similar results were also shown by analyzing rat bmMSCs (Supplementary Figure 1). Since AG1024 targets IGF-IR auto-phosphorylation for down-regulation and phosphorylation at the Ser1135 and Ser1136 sites of IGF-IR activates the receptor kinase, we examined if aging decreased the Ser1135/1136-phosphorylation of IGF-IR. We treated serum-starved Adult-1 and Aged-1 bmMSCs with 0, 50, and 250 ng/ml of IGF-I for 0, 5, 10, and 20 min. Western blot analyses showed that Ser1135/1136-phosphorylated IGF-IR was barely detected in the serum-starved, untreated Adult-1 and Aged-1 bmMSCs. Treatment with 50 ng/ml of IGF-I for 5 min caused approximately 150% and 40% increase of phosphorylation in Adult-1 and Aged-1 cells, respectively, while treatment with 250 ng/ml of IGF-I for 5 min caused approximately 130% and 150% increase of phosphorylation in Adult-1 and Aged-1 cells, respectively (Figure 2B). The IGF-IR levels decreased with increasing IGF-I doses and with time in both types of cells, which might be due to the internalization and subsequent degradation of the IGF-I-IGF-IR complex. So, IGF-IR auto-phosphorylation in response to IGF-I was impaired in Aged-1 cells, whereas this impairment was counteracted by high doses of IGF-I. These results were consistent with those shown in Figure 1A, suggesting that aging inhibited IGF-IR activation and down-regulated the mitogenic activity of bmMSCs in response to IGF-I. The aging-related impairment in IGF-IR auto-phosphorylation was also seen with rat bmMSCs (Supplementary Figure 2).

### Effect of DCN on the DNA synthesis and the auto-phosphorylation of IGF-IR of human bmMSCs

**Figure 3:**
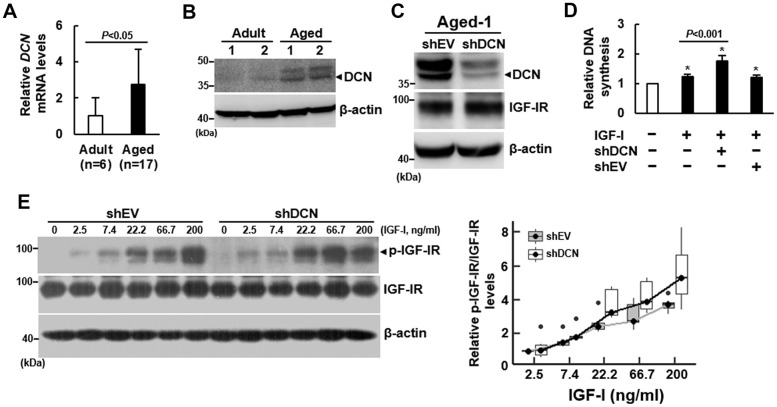
**Effect of DCN knockdown on the DNA synthesis and IGF-IR auto-phosphorylation of aged bmMSCs.** (**A**) RT-qPCR analyses. The expression of *DCN* mRNA of bmMSCs from 17 aged donors was compared to that of the cells from 6 adult donors (to which a value of 1 was assigned). Data represent the mean ± S.D. from a triplicate analysis. Student’s t-test was used to analyze the differences between the groups. (**B**) Western blot analyses of DCN levels. Representative blots of the DCN levels in the Aged-1, Aged-2, Adult-1, and Adult-2 cells are shown. (**C**) Western blot analysis of DCN and IGF-IR levels in cells with DCN knockdown. Aged-1 cells were infected with Lenti virus to generate DCN-knockdown (chDCN) and empty vector control (shEV) cells. The DCN and IGF-IR protein levels of the parental Aged-1, shDCN, and shEV cells are shown. (**D**) BrdU incorporation analyses. Serum-starved parental, shDCN, and shEV cells were treated with 200 ng/ml IGF-I for 24h, and examined for the IGF-I-induced DNA synthesis. The DNA syntheses in these cells were compared to that of the untreated parental cells (to which a value of 1 was assigned). Data represent the mean ± S.D. from three experiments. A one-way ANOVA plus Scheffe’s post hoc tests were used to analyze the differences among the untreated and IGF-1-treated groups. ^*^, *P*<0.05 versus untreated control. Student’s t-test was used to analyze the differences between the groups. (**E**) Western blot analyses of IGF-IR auto-phosphorylation. Serum-starved shDCN and shEV cells were either treated with varying doses of IGF-I for 5 min or left untreated. IGF-IR auto-phosphorylation was examined and normalized to total IGF-IR expression. The difference in the response rates between shDCN and shEV cells was analyzed by linear regression analyses.

Based on our previous finding that the expression of *DCN* mRNA in human bmMSCs was increased with advancing age (r=0.57) [5], we examined if DCN played a role in the impairment of IGF-IR activation in response to IGF-I by aging. Consistent with previous findings, RT-qPCR analyses showed that the *DCN* mRNA levels in bmMSCs from aged donors was approximately 2.7 fold of that in bmMSCs from adult donors (Figure 3A), while the DCN protein levels in Adult-1, Adult-2, Aged-1, and Aged-2 cells also showed that DCN expression increased with aging (Figure 3B). To examine the role of DCN in the IGF-I signaling of bmMSCs, we knocked down DCN expression (approximately 52%) in Aged-1 cells, and examined the IGF-I-induced DNA synthesis. DCN knockdown did not affect IGF-IR expression (Figure 3C). BrdU incorporation assays showed that 200 ng/ml of IGF-I caused approximately 22% (*P*<0.05), 75% (*P*<0.05), and 20% (*P*<0.05) increase in DNA synthesis in the parental, DCN-knockdown (shDCN), and empty-vector control (shEV) Aged-1 cells, respectively (Figure 3D). So, DCN knockdown significantly increased IGF-I-induced DNA synthesis (*P*<0.001). Consistently, DCN knockdown enhanced IGF-I-induced DNA synthesis in Aged-2 cells (Supplementary Figure 3). Subsequently, we performed dose-response experiments to examine the effects of DCN knockdown on IGF-IR auto-phosphorylation. We treated serum-starved shEV and shDCN cells with or without increasing doses of IGF-I for 5 min, and examined the phosphorylated-IGF-IR-to-IGF-IR ratios (Figure 3E). We used linear regression to analyze the response rates of shEV and shDCN cells. The correlation coefficients between IGF-I concentrations and IGF-IR auto-phosphorylation were 0.4 (*P*=0.005) for shEV, and 0.7 (*P*<0.0001) for shDCN, indicating that the IGF-IR auto-phosphorylation in both cells increased significantly in response to increasing IGF-I doses, and that the response rate of shDCN cell was significantly higher than that of shEV cells (Figure 3E). On the other hand, IGF-I seemed not to modulate DCN expression in both groups of cells (Supplementary Figure 4A).

**Figure 4:**
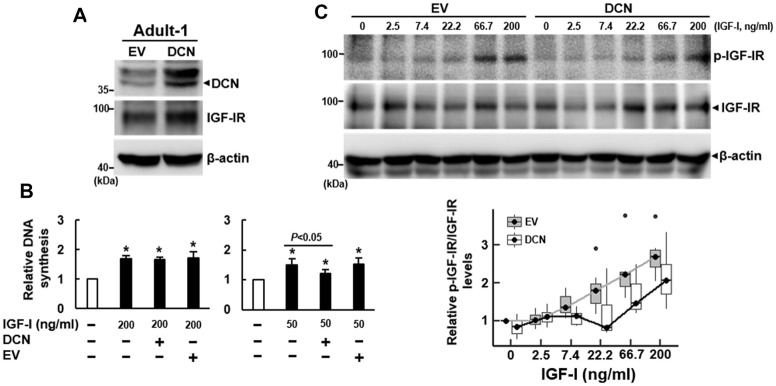
**Effect of DCN overexpression on the DNA synthesis and IGF-IR auto-phosphorylation of adult bmMSCs.** (**A**) Western blot analyses of DCN and IGF-IR levels in cells overexpressing DCN. Adult-1 cells were infected with Lenti virus to generate DCN-overexpressing (DCN) and empty vector control (EV) cells. The DCN and IGF-IR protein levels of the parental Aged-1, DCN, and EV cells are shown. (**B**) BrdU incorporation analyses. Serum-starved parental, DCN, and EV cells were examined for DNA synthesis induced by IGF-I (50 and 200 ng/ml). The DNA syntheses in these cells were compared to that of the untreated parental cells (to which a value of 1 was assigned). Data represent the mean ± S.D. from three experiments. A one-way ANOVA plus Scheffe’s post hoc tests were used to analyze the differences among the untreated and IGF-1-treated groups. ^*^, *P*<0.05 versus untreated control. Student’s t-test was used to analyze the differences between the groups. (C) Western blot analyses of IGF-IR auto-phosphorylation in cells overexpressing DCN. Serum-starved DCN and EV cells were either treated with varying doses of IGF-I for 5 min or left untreated, and the IGF-IR auto-phosphorylation in response to IGF-I was examined by Western blot analyses. The difference in the response rates between DCN and EV cells was analyzed by linear regression.

We also overexpressed DCN (approximately 68%) in Adult-1 cells (Figure 4A), and examined the IGF-I-induced DNA synthesis. DCN overexpression seemed not to affect IGF-IR expression (Figure 4A). BrdU incorporation assays showed that 200 ng/ml of IGF-I caused approximately 69% (*P*<0.05), 66% (*P*<0.05), and 72% (*P*<0.05) increase in DNA synthesis in the parental, DCN-overexpressing (DCN), and empty-vector control (EV) Adult-1 cells, respectively, and DCN overexpression seemed not to decrease DNA synthesis (Figure 4B, left). Considering that high dose of IGF-I might counteract the inhibitory effect of DCN overexpression (68%) on DNA synthesis, we decreased IGF-I dose. As a result, 50 ng/ml of IGF-I caused approximately 50% (*P*<0.05), 21% (*P*<0.05), and 52% (*P*<0.05) increase in DNA synthesis in those cells, respectively, and DCN overexpression significantly decreased the IGF-I-induced DNA synthesis (*P*<0.05) (Figure 4B, right). Consistently, IGF-I (50 ng/ml) induced DNA synthesis in Adult-2 cells, which was decreased by DCN overexpression (Supplementary Figure 3). To examine the rates of IGF-IR auto-phosphorylation of control (EV) and DCN-overexpressing (DCN) Adult-1 cells in response to IGF-I, we treated these serum-starved cells with IGF-I as described for the shEV and shDCN cells (Figure 3E). IGF-I seemed not to modulate DCN expression in both groups of cells (Supplementary Figure 4B). Linear regression analysis revealed no significant difference in the response rates between EV and DCN cells. However, if the data from cells treated with 200 ng/ml IGF-I were excluded from the comparison, the correlation coefficients between IGF-I concentrations and IGF-IR auto-phosphorylation were 0.35 (*P*<0.0001) for EV cells and -0.17 (*P*=0.024) for DCN cells. These data indicated that the IGF-IR auto-phosphorylation in both cells increased significantly in response to increasing IGF-I doses, and that the response rate of DCN cell was significantly lower than that of EV cells (Figure 4C). Taken together, our data indicated that DCN acted as an inhibitor of IGF-IR of human bmMSCs.

### Effect of IGF-I on the binding of DCN to IGF-IR

**Figure 5:**
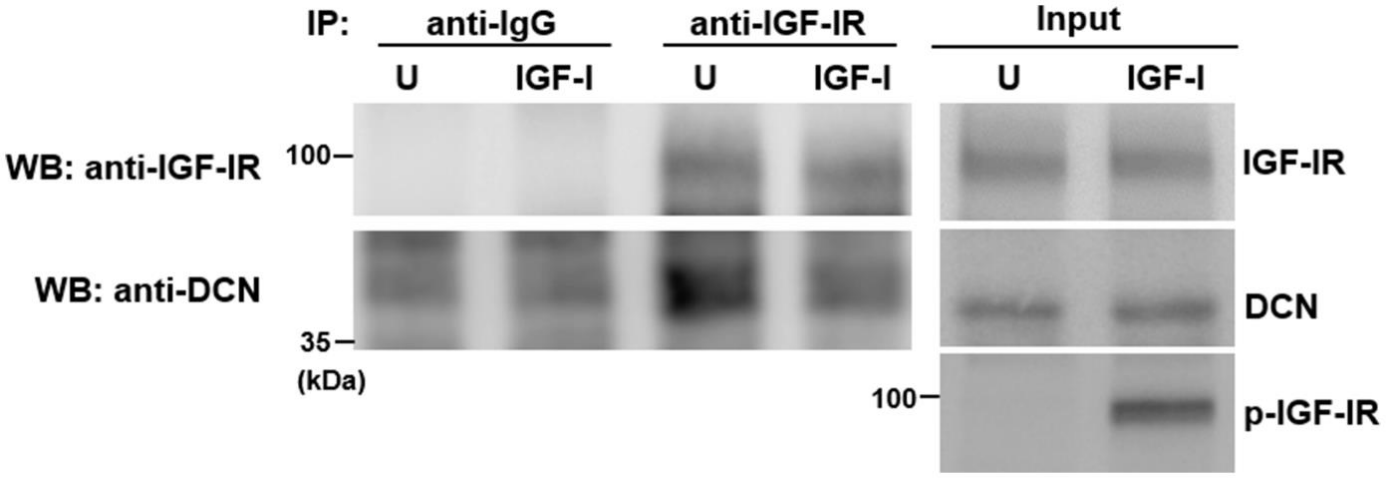
**Effect of IGF-I on the binding of DCN to IGF-IR.** Aged-1 cells were serum-starved for 16 h, and then either treated with 200 ng/ml IGF-I (IGF-I) for 5 min or left untreated (U). Cells were harvested for co-immunoprecipitation assays and Western blot analyses for DCN and IGF-IR.

To examine how IGF-I counteracted the inhibitory effect of DCN on IGF-IR, we performed immunoprecipitation and Western blot analyses on serum-starved Aged-1 cells with or without IGF-I treatment to examine if DCN interacted with IGF-IR, and if IGF-I decreased the binding of DCN to IGF-IR. Results showed that a substantial amount of DCN was co-precipitated with IGF-IR, and that IGF-I add-back (250 ng/ml, 5 min) induced IGF-IR auto-phosphorylation and decreased the binding of DCN to IGF-IR, but did not increase IGF-IR levels in the input and precipitates (Figure 5). These data suggested that IGF-I and DCN might bind to the IGF-IR of human bmMSCs in a competitive manner. We also examined the co-precipitation of DCN with IGF-IR in Adult-1 and Aged-1 cells, and found that IGF-IR in Aged-1 cells bound more DCN as compared with that of Adult-1 cells (Supplementary Figure 5).

### DNA methylation regulated DCN expression

**Figure 6:**
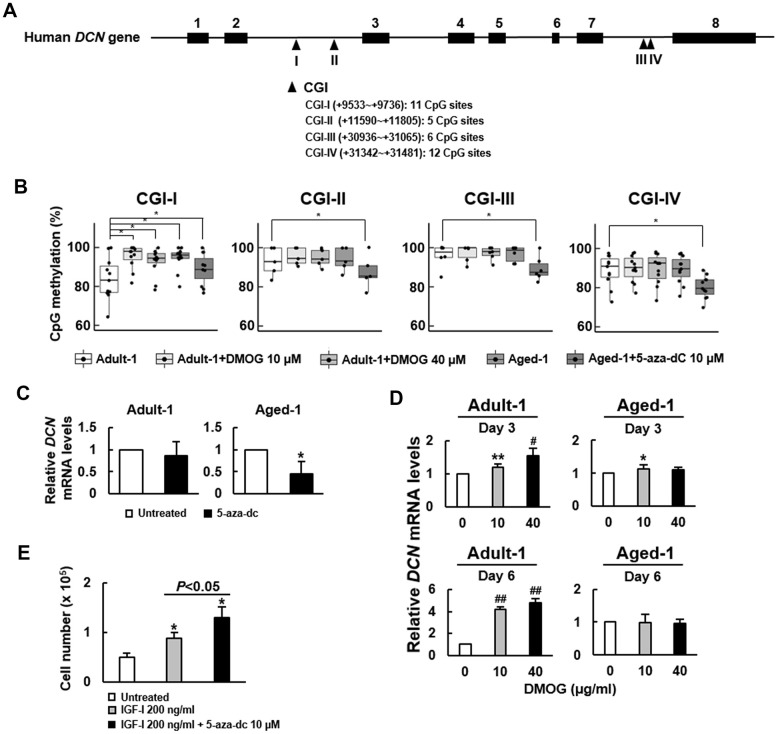
**Effect of 5-aza-dC and DMOG on the methylation of CpG islands in *DCN* gene and the expression of *DCN* mRNA in Adult-1 and Aged-1 bmMSCs.** (**A**) Schematic presentation of human *DCN* gene. Eight exons (■) and 4 predicted CpG islands (▲) in the introns are shown. CGI-I, -II, -III, and -IV contains 11, 5, 6, and 12 CpG sites, respectively. (**B**) Bisulfite sequencing. Adult-1 and Aged-1 cells were treated with DMOG (10 and 40 μM) and 5-aza-dC (10 μM) for 6 days, respectively. The methylation percentage of each CpG site of the 4 CGIs was determined. A one-way ANOVA plus Scheffe’s post hoc tests were used to analyze the differences among the treated Adult-1 and Aged-1 groups versus untreated Adult-1 cells. ^*^, *P*<0.05 versus untreated Adult-1 cells. (**C**) RT-qPCR analyses. Adult-1 and Aged-1 cells were either left untreated or treated with 10 μM 5-aza-dC for 6 days. *DCN* mRNA levels of the treated cells were compared to those of the untreated. Data represent the mean ± S.D. from three experiments. ^*^, *P*<0.01 versus untreated control by Student’s t-test. (**D**) RT-qPCR analyses. Adult-1 and Aged-1 cells were treated with 0, 10, and 40 μM DMOG for 3 and 6 days, and the *DCN* mRNA levels were measured. The normalized *DCN* signals in these cells were compared to that of the untreated cells (to which a value of 1 was assigned). Data represent the mean ± S.D. from three experiments. ^*^, *P*<0.05; ^**^, *P*<0.005; ^#^, *P*<10^-4^; ^##^, *P*<10^-6^ versus untreated control by Student’s t-test. (**E**) Proliferation assay. Aged-1 cells were seeded into 12 10-cm cell culture dishes (1 x 10^5^ cells/dish) and were serum-starved for 16h. Then, 4 dishes of cells were left untreated, 4 dishes of cells were treated with 200 ng/ml IGF-I, and the other 4 dishes were treated with 200 ng/ml IGF-I plus 10 μM of 5-aza-dC. Cells were counted 6 days after. Data represent the mean ± SD. ^*^, *P*<0.05 versus the untreated group. A one-way ANOVA plus Scheffe’s post hoc tests were used to analyze the differences among the groups. Student’s t-test was used to analyze the difference between the IGF-I-treated and the IGF-I plus 5-aza-dC-treated groups.

Epigenetic regulation is an important mechanism underlying the gene expression in aging, and has been shown to regulate DCN expression in non-small cell lung cancer cells [19]. To assess if DNA methylation plays a role in regulating DCN expression in bmMSCs, we treated adult and aged rat bmMSCs with or without 5-aza-2’-deoxycytidine (5-aza-dC, a DNA methylation inhibitor) and dimethyloxalylglycine (DMOG, an inhibitor of the ten-eleven translocation proteins (TETs) that cause DNA demethylation) for 3 and 6 days, and then examined *Dcn* mRNA expression. The results showed that 5-aza-dC (10 μM) decreased *Dcn* expression in aged bmMSCs, whereas DMOG (10 μM) increased *Dcn* expression in adult bmMSCs (Supplementary Figure 6). These data suggested a positive association between DNA methylation and *Dcn* expression. Subsequently, to examine if these results were also seen with human bmMSCs, first, we analyzed the *DCN* gene sequence using the online MethPrimer for the clusters of cysteine-guanine dinucleotides (CpGs), and located 4 CpG islands (CGIs) in the second and seventh introns, containing 11, 5, 6, and 12 CpG sites, respectively (Figure 6A). We treated Adult-1 and Aged-1 cells with DMOG (10 and 40 μM) and 5-aza-dC (10 μM) for 6 days, respectively, and measured the methylation status of those 34 CpG sites (Supplementary Table 1). We found that the median of methylation of Adult-1 and Aged-1 cells was 83.1% and 96.1%, respectively, in CGI-I; 92.7% and 92.9%, respectively, in CGI-II; 97.7% and 98.6%, respectively, in CGI-III; and 89.2% and 89.1%, respectively, in CGI-IV (Figure 6B). Notably, the *DCN* methylation of Aged-1 cells was significantly higher than that of Adult-1 cells only in CGI-I (*P*<0.05). Also, DMOG dose-dependently increased DNA methylation of Adult-1 cells (*P*<0.05), whereas 5-aza-dC decreased DNA methylation of Aged-1 cells (*P*<0.05) only in CGI-I. In parallel, both Adult-1 and Aged-1 cells were treated with 10 μM of 5-aza-dC for 6 days. RT-qPCR analyses showed that *DCN* mRNA expression in Adult-1 cells was not significantly changed (*P*=0.299), whereas there was approximately 53% decrease in Aged-1 cells (*P*<0.05) (Figure 6C). Then, both types of cells were treated with 10 μM and 40 μM of DMOG for 3 and 6 days. RT-qPCR analyses showed that *DCN* mRNA expression in Adult-1 cells was significantly increased with increasing DMOG dose and treatment time, whereas there was no significant change in Aged-1 cells except that 10 μM for 3 days increased *DCN* expression slightly (Figure 6D). These data suggested a positive association between DNA methylation and *DCN* expression in human bmMSCs. To examine if inhibition of DNA methylation promoted cell proliferation, we treated serum-starved Aged-1 cells (1 x 10^5^) with or without 10 μM of 5-aza-dC in the presence of IGF-I for 6 days, and compared their cell number with that of the control. The cell number of the control, IGF-I treated, and IGF-I plus 5-aza-dC-treated groups was 5 x 10^4^, 8.9 x 10^4^, and 1.3 x 10^5^, respectively (Figure 6E). These data suggested that IGF-I plus 5-aza-dC increased cell proliferation (Figure 6E).

## DISCUSSION

IGF-I and bmMSCs are two critical components in maintaining bone homeostasis. IGF-I can facilitate the proliferation and osteoblastic differentiation of bmMSCs to produce bone cells to replace the old and damaged bone tissues. However, IGF-I signaling in bmMSCs is impaired by aging. Previous gene expression profiling has revealed a list of genes whose expression increases/decreases in human bmMSCs with advancing age. Here, from those age-associated genes, we have found a potential DCN-based mechanism underlying the aging-related impairment in the mitogenic activity of bmMSCs in response to IGF-I [11, 13].

Theoretically, the defect in the transduction of mitogenic signals of a growth factor may originate from the loss of receptors, the compromised ligand-binding and activation of receptors, and the disintegration of the intracellular portion of the signaling cascade. Studies from us and others have found that the expression of IGF-IR and the binding affinity of IGF-I to IGF-IR are not altered by aging (Figure 2 and Supplementary Figure 2) [11, 17]. Notably, AG1024, which inhibits the auto-phosphorylation of IGF-IR and IR with IC_50_ values around 7 μM and 57 μM, respectively [20], blocked the DNA synthesis as stimulated in the serum-starved aged bmMSCs by high dose of IGF-I (Figure 1) at the dose of 1 μM (Figure 2). These results revealed that high dose of IGF-I stimulates DNA synthesis by stimulating IGF-IR signaling, that the architecture of the intracellular portion of IGF-I signaling cascade may remain integrated in the aged bmMSCs, and most importantly that IGF-IR auto-phosphorylation is the prime target of aging in down-regulating the IGF-I signaling of bmMSCs.

The crucial findings of the age-associated nature of the impairment of IGF-I signaling (Figure 1 and Supplementary Figure 1) and bmMSC’s proliferation rate [5] led us to search from the extracellular glycoproteins for the inhibitors of bmMSC’s IGF-IR auto-phosphorylation. DCN, biglycan (BGN), and lumican (LUM) are ECM proteoglycans whose expression increases with advancing age in human bmMSCs [5]. Among them, DCN has been found to regulate IGF-I signaling in cells other than bmMSCs [21, 22]. Our data showed that DCN knockdown enhanced the IGF-I-induced DNA synthesis and IGF-IR auto-phosphorylation in aged bmMSCs, whereas DCN overexpression inhibited those activities in adult bmMSCs (Figures 3, 4), and that DCN bound to IGF-IR but the binding is inhibited by high dose of IGF-I (Figure 5). These results thus support DCN as an IGF-IR inhibitor that competes with IGF-I to mediate aging’s inhibitory effect on the IGF-I signaling in bmMSCs. Previously, Schaefer et al. examined the binding of human recombinant DCN and IGF-I to the IGF-IR purified from normal rat kidney fibroblasts, and reported that DCN competes with IGF-I but also functions as an IGF-IR activator [21]. However, Iozzo et al. examined the interaction between human recombinant DCN, IGF-I, and IGF-IR, and reported that DCN binds to IGF-IR in the region that does not overlap with the canonical binding site for IGF-I, and that DCN acts as an antagonist of IGF-IR in urothelial cancer cells [22]. Therefore, whether DCN competes with IGF-I to bind to IGF-IR, and whether the binding activates or inactivates IGF-IR may depend on the cell types. Our data showed that aged bmMSCs bound more DCN than adult bmMSCs did (Supplementary Figure 5). Given the data that DCN expression was up-regulated in aged bmMSCs, it is currently unclear if aging increased the binding affinity between DCN and IGF-IR, resulting in the stronger DCN-binding activity by the IGF-IR of aged cells. Nor is it clear if the increased DCN-binding activity by the IGF-IR of aged cells was simply resulted from the fact that there were more DCN molecules for the IGF-IR of aged cells to bind.

Epigenetic mechanisms are well correlated with aging and the age-related decline in tissue function [23]. DNA methylation is one of the epigenetic mechanisms that can either repress or enhance gene expression depending on the genomic regions [reviewed in reference [24]]. In general, methylation of CpGs in the promoter regions, the first exon, and the first intron represses gene expression, whereas methylation in the gene body enhances gene expression. The epigenetic regulation of DCN expression in bmMSCs has not been well addressed. Our analysis did not retrieve CGIs in the promoter, exon, and the first intron regions of *DCN* gene, probably due to the different analytical software used to predict the locations of potential CGIs. Our data revealed an enhancing role DNA methylation played in DCN expression by showing that 5-aza-dC decreased methylation and *DCN* mRNA expression in aged bmMSCs, whereas DMOG increased methylation and *DCN* mRNA expression in adult bmMSCs (Figure 6 and Supplementary Figure 6). While the effect of 5-aza-dC and DMOG can be genome wide, our data showed a positive correlation between the DCN expression and the methylation of several CpG sites in the CGI-I region of *DCN* gene (Supplementary Table 1 and Figure 6B). It is conceivable that CGI-I might overlap with an intragenic suppressor in the second intron of *DCN* gene; decrease and increase of CGI-I methylation by 5-aza-dC and DMOG might concomitantly activate and repress the suppressor, resulting in the decrease and increase of *DCN* mRNA expression, respectively. Interestingly, CGI-I methylation was higher in Aged-1 than in Adult-1 bmMSCs (Figure 6B), and aged bmMSCs expressed more DCN than adult bmMSCs (Figure 3A). The difference in CGI-I methylation between the bmMSCs from another 5 adult and aged donors was, however, not as prominent, and the percent average of methylation of those adult bmMSCs was higher than Adult-1 cells (data not shown). Firstly, it is unclear if CpGs in the CGI-I region of *DCN* gene act as methylation hot-spots for regulating its expression in aging. Secondly, global gene expression profiling has shown that aging and senescence in cultures have related effects on human bmMSCs [25], and our cells have been passaged for several times, and may have experienced different degree of aging in culture. Therefore, while the role of CGI-I methylation in DCN expression in aging requires further investigation, our data suggested a close association between aging, DNA methylation status, and DCN expression in human bmMSCs. Moreover, the proliferation of serum-starved aged bmMSCs in response to IGF-I was further enhanced by 5-aza-dC (Figure 6E), which supported a causative role of increased DNA methylation and hence DCN expression in the impairment of IGF-I signaling in human bmMSCs.

Our study was conducted using serum-starved cultures because serum contains a spectrum of growth factors, and DCN also participates in the signaling such as TGF-β [26], EGF [27], PDGF [28], and BMP4 [29], which may mislead data interpretation. This experimental design deterred our attempt to investigate if DCN knockdown further sensitizes bmMSCs to the pro-osteoblastic effect of IGF-I because cells cannot survive long enough in the IGF-I-only medium for us to detect calcium precipitation by Alizarin Red S staining. Interestingly, it was reported that *Dcn^-/-^* mice did not show bone defect [30]. While this result might suggest no role of DCN in bone formation, however, it could simply mean that DCN is not involved in the developmental stage of skeleton, and it does not exclude a potential role of DCN in mediating aging-related bone loss. To address DCN’s bone effect, it is necessary to conduct histomorphometric analyses on the bones of the aged *Dcn^-/-^* and wild-type mice to see if the aged *Dcn^-/-^* mice have more bone mass than the aged wild-type mice. Informatively, we found that DCN knockdown decreased the adipogenic potential of aged human bmMSCs cultured in full-serum medium (data not shown). This set of data is consistent with the evidence that aged bones contain more adipocytes and less osteoblasts than adult bones. On the other hand, the contributory role of BGN in bone formation has been demonstrated in transgenic mice, and *Bgn^-/-^* mice did show bone defect [30]. However, comparative analysis of the bone mass of aged wild-type and *Bgn^-/-^* mice has not been reported, and we found that *Bgn* expression is up-regulated in bmMSCs of aged donors (data not shown). As BGN plays a role in skeletal development, it will be interesting to examine if it also plays a regulatory role in the aging of bone.

In conclusion, we have discovered an age-associated impairment in the mitogenic activity of bmMSCs in response to IGF-I, and a potential underlying mechanism, where DCN is up-regulated by aging and functions as an IGF-IR inhibitor. DCN expression was found to be positively associated with the methylation of CpGs in the second intron of the gene. However, whether aging increases DCN expression via the methylation of those CpGs requires further investigation. Notably, our data depicted an example to address the potential involvement of glycoproteins in the aging of bmMSCs and perhaps the bones.

## MATERIALS AND METHODS

### Cell culture

F344 rat bmMSCs were a kind gift from Dr. Chun-Chin Liang who had retired from the National Health Research Institutes in Taiwan years ago. Human bmMSCs were prepared from bone marrow samples collected from patients receiving total knee replacement surgery as described previously [5]. Informed consent was obtained from each donor. The use of human bmMSCs was approved by the Institutional Review Board of National Taiwan University Hospital, Hsin-Chu Branch (IRB No. 103-012-F). Flow cytometric analyses were performed using a Becton Dickinson FACS Calibu flow cytometer, and cells that were CD31- and CD45-negative, but CD90- and CD105-positive were recognized as bmMSCs. Both of rat and human bmMSCs were cultured in Dulbecco's modified Eagle's medium (DMEM) (low glucose) (Thermo Fisher Scientific, MA, USA) containing fetal bovine serum (15%) (HyClone Laboratories Inc., UT, USA), glutamine, penicillin and streptomycin (Thermo Fisher Scientific, MA, USA), and maintained in a humidified atmosphere containing 5% CO_2_ at 37° C. Cell culture media were changed every 4 days. Cells cultured between the fourth and seventh passage were used in this study.

### Plasmid construction, Lentivirus preparation, and infection

Human DCN cDNA was amplified by PCR. The 5’ and 3’ primers used were GGAATTCATGAAGGCCACTATCATCCTC and GGAATTCGAATTACTTATAGTTTCCGAGTTG. The cDNA was cloned into pLAS3w.Pneo vector to generate pLAS3w.Pneo-DCN for Lentivirus preparation. pLAS3w.Pneo and pshRNA_DCN_ (clone ID: TRCN0000058556, a plasmid harboring shRNA targeting human *DCN* mRNA with the sequence CCGGCCGTTTCAACAGAGAGGCTTACTCGAGTAAGCCTCTCTGTTGAAACGGTTTTTG) were purchased from the National RNAi Core Facility at Academia Sinica, Taiwan. For DCN knockdown or overexpression in human bmMSCs, pshRNA_DCN_ or pLAS3w.Pneo-DCN was co-transfected with gag-pol and VSV-G-expressing plasmids into 293T cells. Viral supernatant was harvested 2 and 3 days after transfection and filtered through 0.45-μm filters. BmMSCs were infected with virus (MOI = 40) for 3 h in the presence of polybrene (8 μg/ml).

### Measurement of DNA synthesis

DNA synthesis was assessed by measuring the incorporation of 5-bromo-2-deoxyuridine (BrdU) into DNA [13]. Briefly, cells were seeded in 96-well culture plates (1200 cells/well), and maintained in medium containing varying doses of IGF-I for 24h at 37° C. BrdU or phosphate buffer saline (as background control) was added to each well during the final 8h of treatment. After fixation, cells were incubated with anti-BrdU antibody for 1h at room temperature, and with substrate solution for 15 min in the dark at room temperature. The signals were quantitated using a spectrophotometric plate reader set at wavelength of 450/540 nm. To examine if high dose IGF-I also activated the other signaling pathways to stimulate DNA synthesis, cells were treated with IGF-I and AG1024 (Sigma Aldrich, MO, USA) concomitantly.

### Western blot analyses

For Western blot analysis, aliquots (40 μg) of whole-cell lysates were separated on 10% SDS-polyacrylamide gels, and electrotransferred onto polyvinylidene membranes. The membranes were incubated with anti-IGF-IR (Cell Signaling Technology, MA, USA), anti-phosphorylated IGF-IR (Cell Signaling Technology, MA, USA), anti-DCN (GTX101250, Genetex, Taiwan), and anti-β-actin (BD Biosciences, CA, USA) antibodies, and the signals were obtained by enhanced chemilluminescence (PIERCE, IL, USA).

### Quantitative real-time PCR (RT-qPCR) analyses

RT-qPCR was performed as described previously [15]. The 5’ and 3’ primers used were as follows: human *IGF-I*, TGCTTCCGGAGCTGTGATCT and TCTGGGTCTTGGGCATGTC; human *IGF-IR*, TCGACATCCGCAACGACTATC and CCAGGGCGTAGTTGTAGAAGAG; human *DCN*, ACTGGGAGATACAGCCATCCA and GTTATAAAAATGAGGGCTTTCTTGAGA; human *β-ACTIN*, AAGTCCCTTGCCATCCTAAAA and ATGCTATCACCTCCCCTGTG. All RT-qPCRs were performed in triplicate on an ABI PRISM 7000 Sequence Detector System. The relative mRNA levels were calculated using the 2^−ΔΔCT^ method, with *β-ACTIN* mRNA as a normalizer.

### Co-immunoprecipitation assay

To examine the association between IGF-IR and DCN, cells (1.6 x 10^6^) were lysed in RIPA buffer at 4° C for 30 min with gentle shaking. Equal aliquot of cell lysate (200 μg) was then mixed with protein A/G-agarose beads that were pre-coated with anti-IGF-IR antibody or IgG, at 4° C for 16 h with gentle shaking. After low-speed centrifugation, the supernatants were discarded, and the agarose beads were washed 4 times with RIPA buffer, and then subjected to Western blot analysis for IGF-IR and DCN.

### DNA methylation analysis

Genomic DNA was isolated from bmMSCs treated with DMOG, or 5-aza-dC, or left untreated, and was sent for bisulfite conversion and methylation analyses (Mission Biotech, Taipei, Taiwan). Briefly, bisulfite treatment of DNA was performed using EpiTect 96 Bisulfite Kit (Qiagen, MD, USA). PCR amplification of the 4 CpG islands in *DCN* gene was performed using PyroMark PCR Kit (Qiagen, MD, USA). The 5’ and 3’ primers for the amplification of CGI-I~IV were as follows: CGI-I, TTGTTATTTAGGTTGGAGTGTAGTG and Biotin-TTCAATTCTAATTCCTCTTCTTTATCT; CGI-II, GAGGTTGGTGGATTATGAGGTTAG and Biotin-AATTTAAATCCTCACTCCAAAACTATAT; CGI-III, ATTTAGGTTGGATTGTATATAATGGTATAA and Biotin-ACTACCTCCTACCACCAACAAAATCTTAA; and CGI-IV, Biotin-TGAGGATATGAGTTTTGTAGGTTAAGAA and AACTAACATAATAAAACCCCCTCTCTA. The biotin-labeled PCR products were captured by Streptavidin-Sepharose HP (GE Healthcare Bio-Sciences Corp. NJ, USA), and were made single-stranded using a Pyrosequencing Vacuum Prep Tool. The sequencing primers were annealed to the single-stranded PCR products, and pyrosequencing was done using the PyroMark Q24 system (Qiagen, MD, USA). The sequencing primers used were as follows: CGI-I, GTTGGAGTGTAGTGG, AGTTGGGATTATAGGTATT, and AGGATGGTTTAGATTTTTTGAT; CGI-II, AGTTAGGTATTGGTGGTA, and AGATGATGTTATTGTATTTTAGG; CGI-III, TGGTATAATTTAGGTTTATTGTAAT, and AGTTTTTAGAGTAGTTGGGA; and CGI-IV, CCCTCTCTACTAAAAATACA, CCCAACTACTCCAAAAACTAAAACA, and ACACCCCACTTCAACC. Quantitation of cytosine methylation was done using the PyroMark Q24 Software.

### Statistical analysis

One-way ANOVA followed by Scheffe’s post hoc tests was used to examine the significance in multiple comparison. *P* value less than 0.05 was considered statistically significant. For the examination of the impact of DCN overexpression and DCN knockdown on the IGF-IR auto-phosphorylation in response to IGF-I, a linear regression with three covariates, including IGF-I concentrations, cells and their interactions, was used to compare the response rates between the DCN-overexpressing and control adult bmMSCs as well as the response rates between the DCN-knockdown and control aged bmMSCs. *P* value less than 0.05 was considered statistically significant.

## Supplementary Material

Supplementary Figures

Supplementary Table 1

## References

[1] Sahin E, Depinho RA. Linking functional decline of telomeres, mitochondria and stem cells during ageing. Nature. 2010; 464:520–28. 10.1038/nature08982 20336134 PMC3733214

[2] Prockop DJ. Marrow stromal cells as stem cells for nonhematopoietic tissues. Science. 1997; 276:71–74. 10.1126/science.276.5309.71 9082988

[3] Pittenger MF, Mackay AM, Beck SC, Jaiswal RK, Douglas R, Mosca JD, Moorman MA, Simonetti DW, Craig S, Marshak DR. Multilineage potential of adult human mesenchymal stem cells. Science. 1999; 284:143–47. 10.1126/science.284.5411.143 10102814

[4] Shuai Y, Liao L, Su X, Yu Y, Shao B, Jing H, Zhang X, Deng Z, Jin Y. Melatonin treatment improves mesenchymal stem cells therapy by preserving stemness during long-term in vitro expansion. Theranostics. 2016; 6:1899–917. 10.7150/thno.15412 27570559 PMC4997245

[5] Jiang SS, Chen CH, Tseng KY, Tsai FY, Wang MJ, Chang IS, Lin JL, Lin S. Gene expression profiling suggests a pathological role of human bone marrow-derived mesenchymal stem cells in aging-related skeletal diseases. Aging (Albany NY). 2011; 3:672–84. 10.18632/aging.100355 21808097 PMC3181167

[6] Verma S, Rajaratnam JH, Denton J, Hoyland JA, Byers RJ. Adipocytic proportion of bone marrow is inversely related to bone formation in osteoporosis. J Clin Pathol. 2002; 55:693–98. 10.1136/jcp.55.9.693 12195001 PMC1769760

[7] Duque G, Rivas D, Li W, Li A, Henderson JE, Ferland G, Gaudreau P. Age-related bone loss in the LOU/c rat model of healthy ageing. Exp Gerontol. 2009; 44:183–89. 10.1016/j.exger.2008.10.004 18992316

[8] Liang CT, Barnes J, Seedor JG, Quartuccio HA, Bolander M, Jeffrey JJ, Rodan GA. Impaired bone activity in aged rats: alterations at the cellular and molecular levels. Bone. 1992; 13:435–41. 10.1016/8756-3282(92)90087-d 1476822

[9] Singh L, Brennan TA, Russell E, Kim JH, Chen Q, Brad Johnson F, Pignolo RJ. Aging alters bone-fat reciprocity by shifting in vivo mesenchymal precursor cell fate towards an adipogenic lineage. Bone. 2016; 85:29–36. 10.1016/j.bone.2016.01.014 26805026 PMC4792752

[10] Moerman EJ, Teng K, Lipschitz DA, Lecka-Czernik B. Aging activates adipogenic and suppresses osteogenic programs in mesenchymal marrow stroma/stem cells: the role of PPAR-gamma2 transcription factor and TGF-beta/BMP signaling pathways. Aging Cell. 2004; 3:379–89. 10.1111/j.1474-9728.2004.00127.x 15569355 PMC1850101

[11] Tanaka H, Liang CT. Mitogenic activity but not phenotype expression of rat osteoprogenitor cells in response to IGF-I is impaired in aged rats. Mech Ageing Dev. 1996; 92:1–10. 10.1016/S0047-6374(96)01793-9 9032750

[12] Huat TJ, Khan AA, Pati S, Mustafa Z, Abdullah JM, Jaafar H. IGF-1 enhances cell proliferation and survival during early differentiation of mesenchymal stem cells to neural progenitor-like cells. BMC Neurosci. 2014; 15:91. 10.1186/1471-2202-15-91 25047045 PMC4117972

[13] Chen CY, Tseng KY, Lai YL, Chen YS, Lin FH, Lin S. Overexpression of insulin-like growth factor 1 enhanced the osteogenic capability of aging bone marrow mesenchymal stem cells. Theranostics. 2017; 7:1598–611. 10.7150/thno.16637 28529639 PMC5436515

[14] Bhaumick B, Bala RM, Hollenberg MD. Somatomedin receptor of human placenta: solubilization, photolabeling, partial purification, and comparison with insulin receptor. Proc Natl Acad Sci USA. 1981; 78:4279–83. 10.1073/pnas.78.7.4279 6270667 PMC319773

[15] Kavran JM, McCabe JM, Byrne PO, Connacher MK, Wang Z, Ramek A, Sarabipour S, Shan Y, Shaw DE, Hristova K, Cole PA, Leahy DJ. How IGF-1 activates its receptor. Elife. 2014; 3:e03772. 10.7554/eLife.03772 25255214 PMC4381924

[16] Werner H, Weinstein D, Bentov I. Similarities and differences between insulin and IGF-I: structures, receptors, and signalling pathways. Arch Physiol Biochem. 2008; 114:17–22. 10.1080/13813450801900694 18465355

[17] Cao JJ, Kurimoto P, Boudignon B, Rosen C, Lima F, Halloran BP. Aging impairs IGF-I receptor activation and induces skeletal resistance to IGF-I. J Bone Miner Res. 2007; 22:1271–79. 10.1359/jbmr.070506 17488198

[18] Schaefer L, Iozzo RV. Biological functions of the small leucine-rich proteoglycans: from genetics to signal transduction. J Biol Chem. 2008; 283:21305–09. 10.1074/jbc.R800020200 18463092 PMC2490788

[19] Qian Q, Shi X, Lei Z, Zhan L, Liu RY, Zhao J, Yang B, Liu Z, Zhang HT. Methylated +58CpG site decreases DCN mRNA expression and enhances TGF-β/Smad signaling in NSCLC cells with high metastatic potential. Int J Oncol. 2014; 44:874–82. 10.3892/ijo.2014.2255 24424784

[20] Párrizas M, Gazit A, Levitzki A, Wertheimer E, LeRoith D. Specific inhibition of insulin-like growth factor-1 and insulin receptor tyrosine kinase activity and biological function by tyrphostins. Endocrinology. 1997; 138:1427–33. 10.1210/endo.138.4.5092 9075698

[21] Schaefer L, Tsalastra W, Babelova A, Baliova M, Minnerup J, Sorokin L, Gröne HJ, Reinhardt DP, Pfeilschifter J, Iozzo RV, Schaefer RM. Decorin-mediated regulation of fibrillin-1 in the kidney involves the insulin-like growth factor-I receptor and mammalian target of rapamycin. Am J Pathol. 2007; 170:301–15. 10.2353/ajpath.2007.060497 17200203 PMC1762680

[22] Iozzo RV, Buraschi S, Genua M, Xu SQ, Solomides CC, Peiper SC, Gomella LG, Owens RC, Morrione A. Decorin antagonizes IGF receptor I (IGF-IR) function by interfering with IGF-IR activity and attenuating downstream signaling. J Biol Chem. 2011; 286:34712–21. 10.1074/jbc.M111.262766 21840990 PMC3186372

[23] Benayoun BA, Pollina EA, Brunet A. Epigenetic regulation of ageing: linking environmental inputs to genomic stability. Nat Rev Mol Cell Biol. 2015; 16:593–610. 10.1038/nrm4048 26373265 PMC4736728

[24] Anastasiadi D, Esteve-Codina A, Piferrer F. Consistent inverse correlation between DNA methylation of the first intron and gene expression across tissues and species. Epigenetics Chromatin. 2018; 11:37. 10.1186/s13072-018-0205-1 29958539 PMC6025724

[25] Wagner W, Bork S, Horn P, Krunic D, Walenda T, Diehlmann A, Benes V, Blake J, Huber FX, Eckstein V, Boukamp P, Ho AD. Aging and replicative senescence have related effects on human stem and progenitor cells. PLoS One. 2009; 4:e5846. 10.1371/journal.pone.0005846 19513108 PMC2688074

[26] Hildebrand A, Romarís M, Rasmussen LM, Heinegård D, Twardzik DR, Border WA, Ruoslahti E. Interaction of the small interstitial proteoglycans biglycan, decorin and fibromodulin with transforming growth factor beta. Biochem J. 1994; 302:527–34. 10.1042/bj3020527 8093006 PMC1137259

[27] De Luca A, Santra M, Baldi A, Giordano A, Iozzo RV. Decorin-induced growth suppression is associated with up-regulation of p21, an inhibitor of cyclin-dependent kinases. J Biol Chem. 1996; 271:18961–65. 10.1074/jbc.271.31.18961 8702560

[28] Nili N, Cheema AN, Giordano FJ, Barolet AW, Babaei S, Hickey R, Eskandarian MR, Smeets M, Butany J, Pasterkamp G, Strauss BH. Decorin inhibition of PDGF-stimulated vascular smooth muscle cell function: potential mechanism for inhibition of intimal hyperplasia after balloon angioplasty. Am J Pathol. 2003; 163:869–78. 10.1016/S0002-9440(10)63447-5 12937128 PMC1868258

[29] Chen XD, Fisher LW, Robey PG, Young MF. The small leucine-rich proteoglycan biglycan modulates BMP-4-induced osteoblast differentiation. FASEB J. 2004; 18:948–58. 10.1096/fj.03-0899com 15173106

[30] Corsi A, Xu T, Chen XD, Boyde A, Liang J, Mankani M, Sommer B, Iozzo RV, Eichstetter I, Robey PG, Bianco P, Young MF. Phenotypic effects of biglycan deficiency are linked to collagen fibril abnormalities, are synergized by decorin deficiency, and mimic Ehlers-Danlos-like changes in bone and other connective tissues. J Bone Miner Res. 2002; 17:1180–89. 10.1359/jbmr.2002.17.7.1180 12102052

