# Supplementary material for: Decorin inhibits the insulin-like growth factor I signaling in bone marrow mesenchymal stem cells of aged humans
Source: Aging (Albany NY). 2020 Nov 26;13(1):578–97. doi: 10.18632/aging.202166 (PMC7835024; doi:10.18632/aging.202166)
Supplement: Supplementary Figures [file aging-13-202166-s001.pdf]

SUPPLEMENTARY MATERIALS

Supplementary Figures

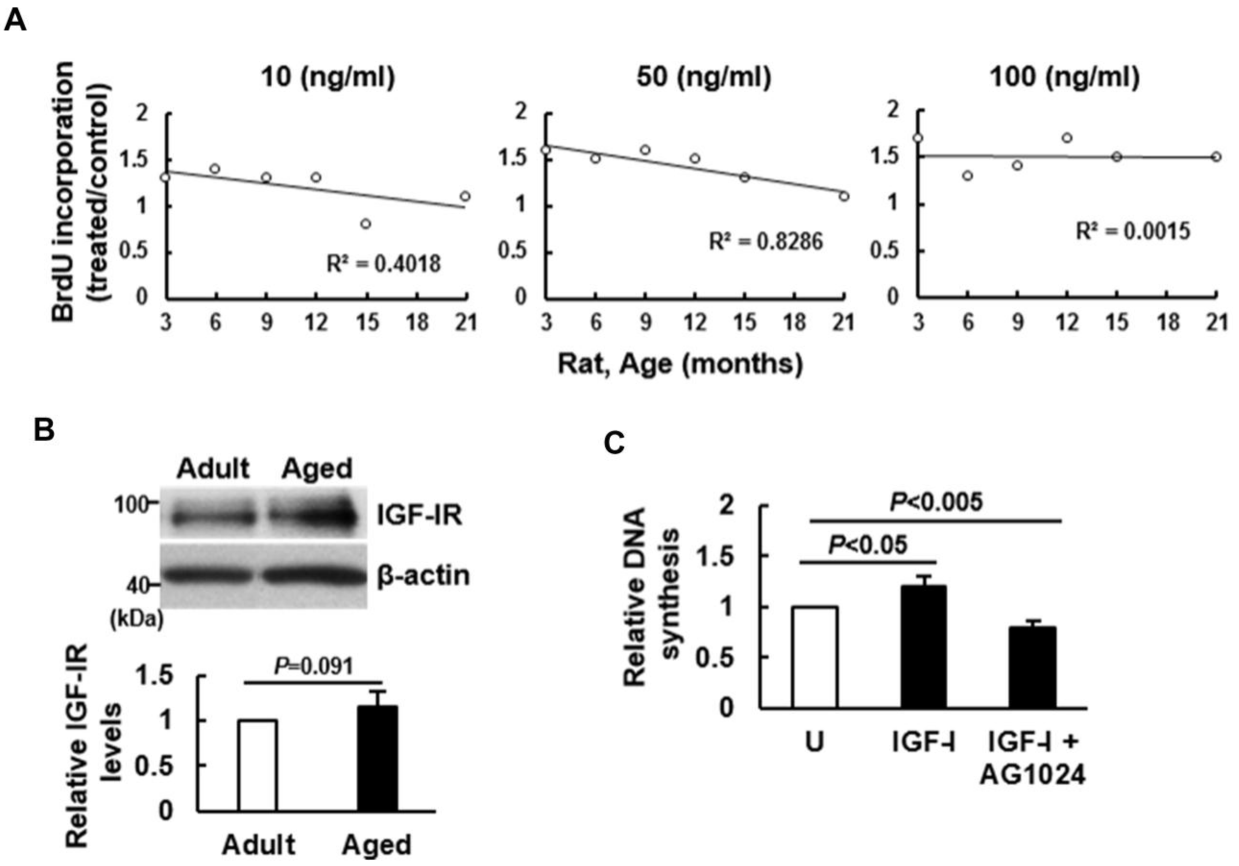

Supplementary Figure 1. Effect of age on the mitogenic response of rat bmMSCs to IGF-I. (**A**) BrdU incorporation analyses. Rat bmMSCs derived from the rats of 3, 6, 9, 12, 15, 18, and 21 months old were maintained in serum-free media for 24 h, and then subjected to BrdU incorporation analyses with or without concomitant treatment of 10, 50, and 100 ng/ml of IGF-I. The correlation between the age and the IGF-I-triggered DNA synthesis was analyzed as described for human bmMSCs. (**B**) Western blot analyses. IGF-IR levels of bmMSCs from adult (6 months) and aged (21 months) rats was measured, and the later was normalized to the former. Student's t-test was used to analyze the difference. (**C**) BrdU incorporation analyses. Serum-starved bmMSCs from the 21-month-old rats were examined for the IGF-I (100 ng/ml)-induced DNA synthesis with or without concomitant treatment with AG1024 (1 μM). Relative DNA synthesis was calculated by compared the OD_{450} readings of the treated cells to that of the untreated (U) cells. Data represent the mean ± S.D. from three experiments. Student's t-test was used to analyze the differences between the groups.

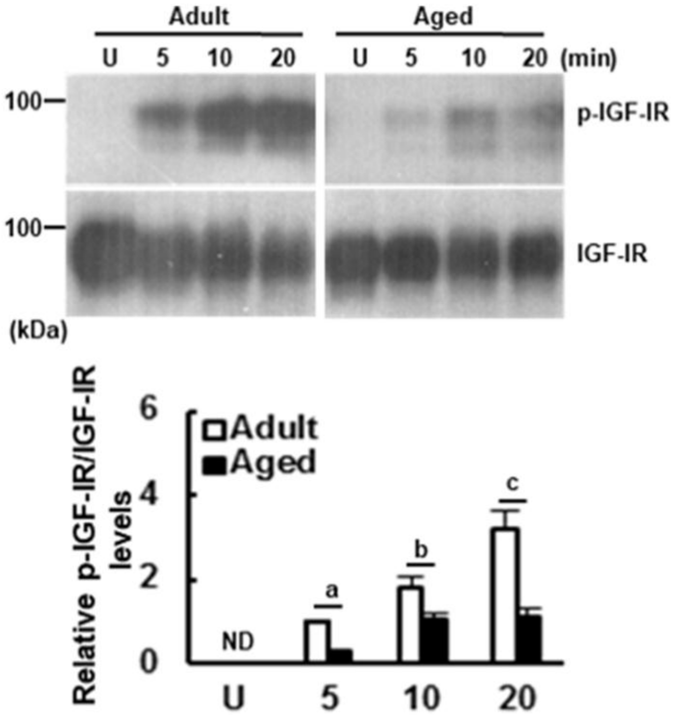

Supplementary Figure 2. Effect of aging on the auto-phosphorylation of IGF-IR of rat bmMSCs. Western blot analyses. Serum-starved aged and adult rat bmMSCs were treated with 100 ng/ml of IGF-I for 0, 5, 10, and 20 min. The auto-phosphorylation of IGF-IR was examined. All the signals were compared to that of the untreated cells (U). Data represent the mean ± S.D. from three experiments. [a], $P<10^{-5}$; [b], $P<0.01$; [c], $P<0.0005$ by Student's t-test.

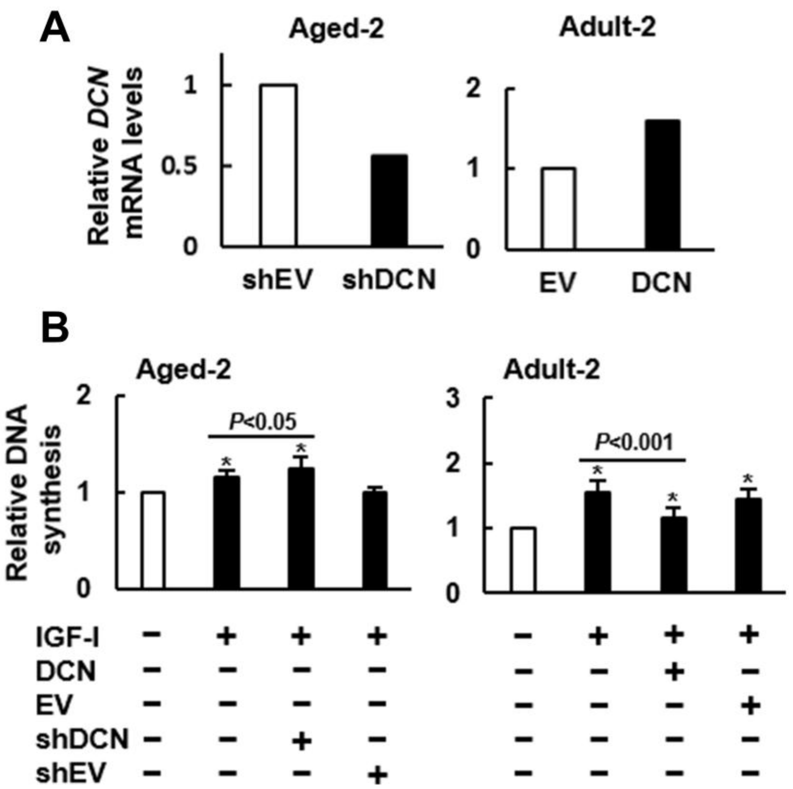

Supplementary Figure 3. Effect of DCN knockdown and overexpression on the DNA synthesis and IGF-IR auto-phosphorylation of aged and adult bmMSCs. (**A**) RT-qPCR analyses. DCN knockdown in human Aged-2 bmMSCs and DCN overexpression in human Adult-2 bmMSCs were examined. (**B**) BrdU incorporation analyses. Serum-starved parental, shDCN, and shEV Aged-2 bmMSCs were treated with 200 ng/ml IGF-I for 24h, and serum-starved parental, DCN, and EV Adult-2 bmMSCs were treated with 50 ng/ml IGF-I for 24h. These cells were then examined for DNA synthesis. The DNA syntheses in these cells were compared to that of the untreated parental cells (to which a value of 1 was assigned). Data represent the mean ± S.D. from three experiments. A one-way ANOVA plus Scheffe's post hoc tests were used to analyze the differences among the untreated and IGF-I-treated groups. *, $P<0.05$ versus untreated control. Student's t-test was used to analyze the differences between the groups.

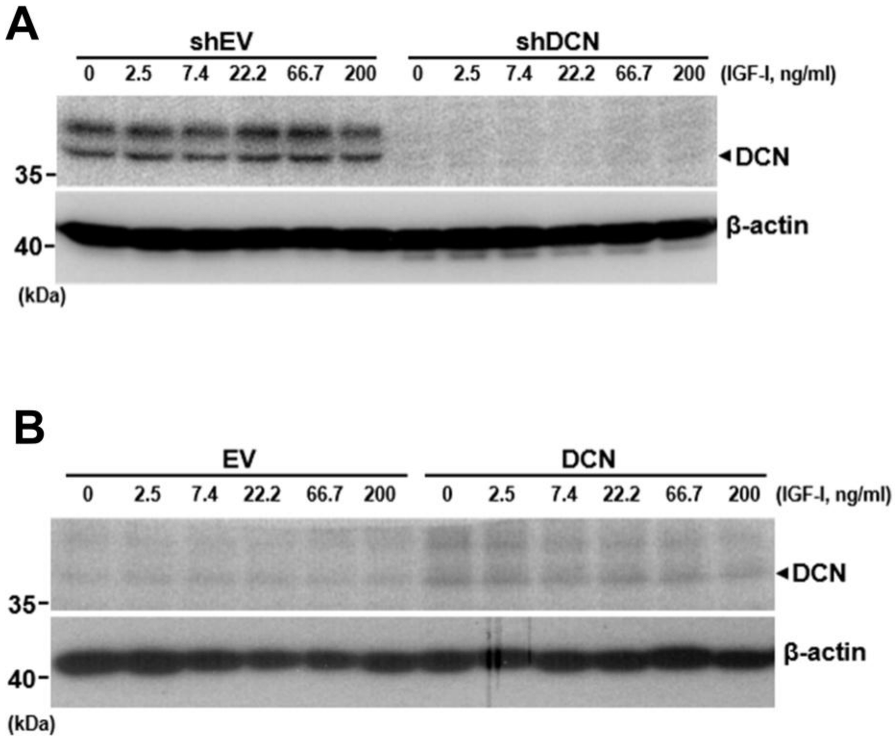

Supplementary Figure 4. Effect of IGF-I on DCN expression in the DCN-knockdown Aged-1 and DCN-overexpressing Adult-1 bmMSCs. Western blot analyses. Serum-starved DCN-knockdown and control Aged-1 (shDCN and shEV) cells (**A**), and serum-starved DCN-overexpressing and control Adult-1 (DCN and EV) cells (**B**) were either treated with increasing doses of IGF-I for 5 min or left untreated. DCN expression was examined.

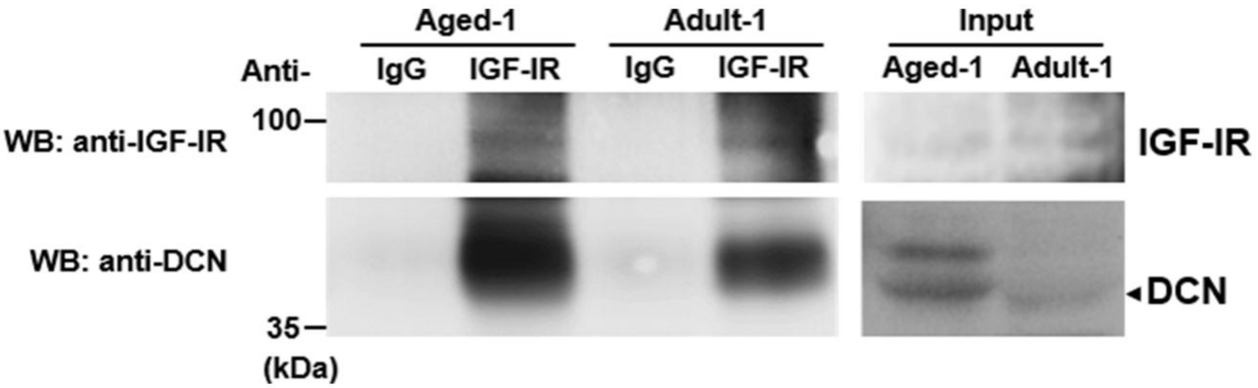

Supplementary Figure 5. Binding of DCN to IGF-IR in Adult-1 and Aged-1 cells. Adult-1 and Aged-1 cells were harvested for co-immunoprecipitation assays and Western blot analyses for DCN and IGF-IR.

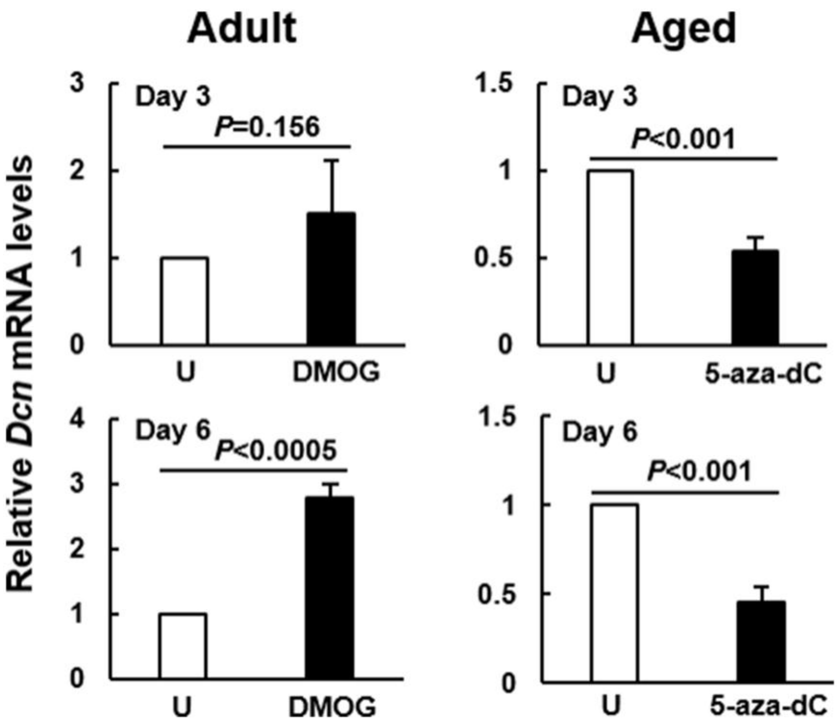

Supplementary Figure 6. Effect of 5-aza-dC and DMOG on the expression of *Dcn* mRNA in adult and aged rat bmMSCs. RT-qPCR analyses. Adult and aged rat bmMSCs were treated with or without 5-aza-dC and DMOG for 3 and 6 days, and the *Dcn* mRNA expression was examined. The results showed that 5-aza-dC (10 μM) decreased *Dcn* expression in the aged bmMSCs, whereas DMOG (10 μM) increased *Dcn* expression in the adult bmMSCs.
