# Supplementary material for: Decorin inhibits the insulin-like growth factor I signaling in bone marrow mesenchymal stem cells of aged humans
Source: Aging (Albany NY). 2020 Nov 26;13(1):578–97. doi: 10.18632/aging.202166 (PMC7835024; doi:10.18632/aging.202166)
Supplement: Supplementary Table 1 [file aging-13-202166-s002.pdf]

SUPPLEMENTARY TABLE

Supplementary Table 1. Effect of aging on the methylation of 4 CpG islands in the DCN gene of human bmMSCs.

CGI-I

CpG site	1	2	3	4	5	6	7	8	9	10	11
Sample ID	Meth. (%)	Meth. (%)	Meth. (%)	Meth. (%)	Meth. (%)	Meth. (%)	Meth. (%)	Meth. (%)	Meth. (%)	Meth. (%)	Meth. (%)
Adult-1	92.18	86.75	99.69	100	88.7	75.56	76.8	83.11	77.46	64.51	77.61
Adult-1 + DMOG 10 μM	95.51	97.91	97.93	100	100	81.81	92.15	100	96.38	86.19	99.24
Adult-1 + DMOG 40 μM	96.67	93.5	98	100	94.95	80.27	94.06	97.35	94.5	90.49	78.43
Aged-1	97.79	94.57	94.58	100	96.94	85.64	94.04	96.15	96.75	79.92	99.8
Aged-1 + 5-aza-dC 10 μM	88.54	90.81	91.22	97.72	88.62	76.82	79.78	97.49	100	88.15	78.69

CGI-II

CpG site	1	2	3	4	5
Sample ID	Meth. (%)	Meth. (%)	Meth. (%)	Meth. (%)	Meth. (%)
Adult-1	92.67	100	99.82	88.36	83.37
Adult-1 + DMOG 10 μM	94.46	100	100	90.66	92.14
Adult-1 + DMOG 40 μM	93.97	100	98.74	89.04	92.26
Aged-1	92.96	100	100	91.01	86.11
Aged-1 + 5-aza-dC 10 μM	85.24	100	90.83	84.77	77.12

CGI-III

Sample ID	Meth. (%)	Meth. (%)	Meth. (%)	Meth. (%)	Meth. (%)	Meth. (%)
Adult-1	95.42	100	100	95.01	100	84.98
Adult-1 + DMOG 10 μM	99.48	100	100	93.73	100	90.04
Adult-1 + DMOG 40 μM	98.05	97.57	100	95.55	100	91.34
Aged-1	97.23	100	100	91.81	100	91.64
Aged-1 + 5-aza-dC 10 μM	86.54	88.23	100	85.72	92.98	82.41

CGI-IV

CpG site	1	2	3	4	5	6	7	8	9	10	11	12
Sample ID	Meth. (%)	Meth. (%)	Meth. (%)	Meth. (%)	Meth. (%)	Meth. (%)	Meth. (%)	Meth. (%)	Meth. (%)	Meth. (%)	Meth. (%)	Meth. (%)
Adult-1	77.45	91.01	72.69	49.94	93.01	92.4	87.31	96.3	78.09	97.48	84.37	86.65
Adult-1 + DMOG 10 μM	83.84	93.59	81.99	48.37	90.29	92.12	88.78	96.48	77.39	97.97	82.73	88.62
Adult-1 + DMOG 40 μM	82.1	92.49	80.65	49.75	93.08	92.47	86.77	97.38	73.38	98.16	82.98	88.52
Aged-1	83.81	92.26	80.32	33.34	92.12	89.66	88.63	97.75	75.75	96.31	83.57	88.1
Aged-1 + 5-aza-dC 10 μM	78.12	84.09	74.98	31.96	79.56	82.81	78.58	87	69.76	88.92	74.6	78.23
